# An In Vitro Evaluation of Anti-inflammatory and Antioxidant Activities of Cocos nucifera and Triticum aestivum Formulation

**DOI:** 10.7759/cureus.48649

**Published:** 2023-11-11

**Authors:** Priyadharshini G, Deepak Pandiar, Rajeshkumar Shanmugam, Reshma Poothakulath Krishnan

**Affiliations:** 1 Oral Pathology and Microbiology, Saveetha Dental College and Hospitals, Saveetha Institute of Medical and Technical Sciences, Saveetha University, Chennai, IND; 2 Pharmacology, Saveetha Dental College and Hospitals, Saveetha Institute of Medical and Technical Sciences, Saveetha University, Chennai, IND

**Keywords:** antioxidant activity, triticum aestivum, cocos nucifera, oral gel, anti-inflammatory

## Abstract

Background

Medicinal plants are traditionally used in Ayurveda, Unani medicine, and Siddha as primary sources of drugs, and mankind has exploited the therapeutic properties of these herbs throughout history. Coconut (*Cocos nucifera*), a common ingredient of Indian sub-continental cuisine, has been proven to possess various medicinal properties; similarly, wheatgrass (*Triticum aestivum*) is of greater medicinal value and is known as the powerhouse of nutrients and vitamins. These have been used individually, but there is limited data on the synergistic use of these products. Thus, the present in vitro study was designed to prepare an oral gel from the extract of *C. nucifera *and *T. aestivum* and to assess its cumulative anti-inflammatory and antioxidant activity.

Materials and methods

*C. nucifera *extract and *T. aestivum *extract were prepared separately, and gel formulation was done. The formulated gel was tested for its anti-inflammatory and antioxidant activity.

Results

The results of the present study demonstrated that the anti-inflammatory property of the gel formulation was greater as compared to the standard (diclofenac), with the highest percentage of inhibition of 90.1% at 50 μl. With regard to the antioxidant property, we found that it was comparable to the standard (ascorbic acid) at various concentrations, with greater activity at 50 μl.

Conclusion

The oral gel formulation of coconut (*C. nucifera*) and wheatgrass (*T. aestivum*) showed better anti-inflammatory and a comparable antioxidant activity. Thus, this formulation may be employed as an adjunct to the commercially available oral gel preparations.

## Introduction

The utilization of natural products for therapeutic purposes is an age-old science, and for a very long time, the primary sources of medicines were minerals and products of various plants and animals [1]. These herbal formulations are the basis of many ancient medicines, including Ayurveda, Siddha, and Unani. Research on the plants and their products or formulations used in traditional medicine has attracted a lot of interest in recent years. Indigenous cultures globally have turned to herbal remedies as a result of the resurgence of interest in medicinal plants [2]. Natural resources are becoming increasingly sought-after as a source for the creation of nutraceuticals, medications, medicines, and various cosmetics.

Inflammation is a complex immune-mediated innate system of defense produced due to physiological disturbances in the body [3]. This process is mediated by chemical mediators in response to various noxious stimuli that cause vasodilation, increased capillary permeability, and escalated vascular supply to the site of injury. The metabolism of arachidonic acid plays a significant role in the chain of events that make up the process of inflammation. Regardless of the etiology, the genesis of pain is mostly inflammatory. Non-steroidal anti-inflammatory drugs (NSAIDs) are still the mainstay of most physicians all over the world for the treatment of any inflammatory reaction and to relieve pain [4]. There are various side effects associated with NSAIDs, viz., significant gastrointestinal upset, gastritis, ulceration, and hemorrhage. The long-term use of these drugs could thus be considered a major health concern, and the search for safer, more economical, and more efficient drugs is now of great interest. It has been found that many herbal plants function similar to NSAIDs, significantly mediating inflammatory pathways [5]. 

Alternatively, antioxidants can counter the harmful effects of free radicals. For faster and more efficient recovery, more antioxidants from food and other sources, including medicinal plants, are needed in diseased conditions [6]. Therefore, it is essential to assess protection against oxidative stress and associated illnesses, and therefore, a different and far more likely antioxidant regimen based on herbal origin would be required, and natural products may be exploited to minimize the side effects of commercially available products.

*Cocos nucifera* (coconut tree) bears various medicinal properties. Coconut milk, an emulsion made from its endosperm, is a rich source of lipids, carbohydrates, and proteins. Besides, it contains several minor chemicals, such as phenolic compounds, and has proven to possess antioxidant, antimicrobial, and anti-inflammatory activities [7]. *Triticum aestivum*, also known as wheatgrass, is an easily grown plant and is considered to be a healthy nutrient food. Studies have proven this plant to have antioxidant and antimicrobial properties owing to its nutritional contents like chlorophyll, vitamin A, vitamin C, and many bioflavonoids.

Previous studies have assessed the medicinal properties of these two herbs separately, but none of the studies have tried to assess their cumulative properties to be used as an oral gel. In our previous study, we formulated an herbal gel combination, and it was proven to have potent antimicrobial activity and less cytotoxic effects [8]. Thus, the present study was orchestrated to analyze and evaluate the antioxidant and anti-inflammatory properties of an oral gel formulation with *C. nucifera* and *T. aestivum* extract and compare the properties with a commercially available oral diclofenac gel. To the best of our knowledge, the herbal combination used in this study is the first of its kind to be used as an oral gel.

## Materials and methods

Preparation of extract and gel

The present in vitro study was designed and conducted in the institutional setting of Saveetha Dental College and Hospitals, Chennai. The endosperm of coconut and wheatgrass were procured from the local market of Chennai for the preparation of extract and gel according to the specifications explained previously [8]. Briefly, 100 mL of distilled water was set to boiling, to which 2 gm of *T. aestivum* (wheatgrass) powder was added. The boiling was carried out for half an hour, followed by filtration through Whatman filter paper. The prepared extract was re-boiled for 10 minutes and finally filtered for further analysis. Regarding *C. nucifera*, 50 gm of fresh coconut was chopped and ground to a smooth paste, to which 100 mL of distilled water was added. This mixture was continuously agitated for one hour, followed by filtration and re-boiling for another 20 minutes.

**Figure 1:**
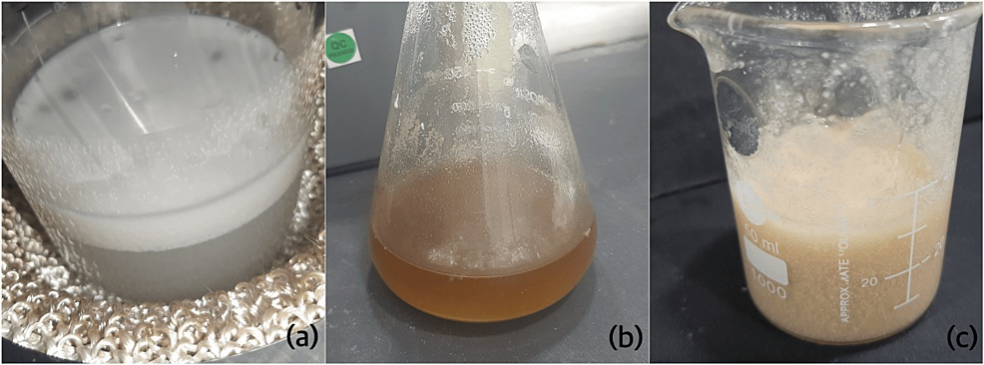
Prepared Cocos nucifera extract (a), Triticum aestivum extract (b), and the gel formulation of Cocos nucifera and Triticum aestivum (c)

For the preparation of the gel formulation, 0.5 gm of hydroxypropyl methylcellulose was mixed with 20 mL of distilled water, to which 0.5 gm of carbachol was added. The mixture was continuously stirred during the entire procedure. This mixture was added to the *C. nucifera* extract, to which *T. aestivum* extract was further added and mixed, yielding the formulation in gel form (Figure 1).

Anti-inflammatory activity assessment using albumin denaturation assay

This assay involves the addition of 2 mL of 1% bovine albumin fraction to 400 mL of herbal extracts of *C. nucifera* and *T. aestivum* at a concentration of 50-150 mL. The pH of the reaction was maintained at 6.8 utilizing 1N hydrochloric acid. The subsequent steps involved incubation at room temperature, followed by heating in a water bath at 55° C for 20 minutes, followed by cooling. The absorbance value was recorded at 600 nm. Diclofenac sodium was used as standard at concentrations of 10 μl, 20 μl, 30 μl, 40 μl, and 50 μl. The percentage inhibition was estimated as % inhibition: (control OD − (sample OD/control OD)) × 100, where control OD designates the absorbance of negative control and sample OD stands for the absorbance of the test sample [9].

Antioxidant activity of the prepared oral gel

The antioxidant capabilities of *C. nucifera*- and *T. aestivum*-based oral gel were tested using the 1,1-diphenyl-2-picrylhydrazyl (DPPH) free radical scavenging assay. The sample, which is an oral gel preparation, was dissolved in methanol at different concentrations (100-500 mg). 3 mL of 0.5 mm DPPH in methanol was added to 0.5 mL of dissolved oral gel preparation and incubated at room temperature for 30 minutes. As a standard control, ascorbic acid was used. The hue of the reaction mixture changed from yellow to violet as oral gel preparation lowered DPPH by donating a hydrogen atom. Ultraviolet-visible (UV-Vis) spectrophotometer was used to assess the absorbance of the mixture at 517 nm: % scavenging activity = ((absorbance of control − absorbance of the test)/absorbance of control) × 100.

Statistical analysis

The obtained values were entered in a Microsoft Excel sheet. The tests were repeated three times, and a mean of the three values was obtained using IBM SPSS Statistics for Windows, Version 26.0 (Released 2019; IBM Corp., Armonk, New York, United States). For the comparison of means between the control and experimental groups at different concentrations, an independent t-test was used. A p-value of less than or equal to 0.05 was considered significant.

## Results

Anti-inflammatory activity

**Figure 2:**
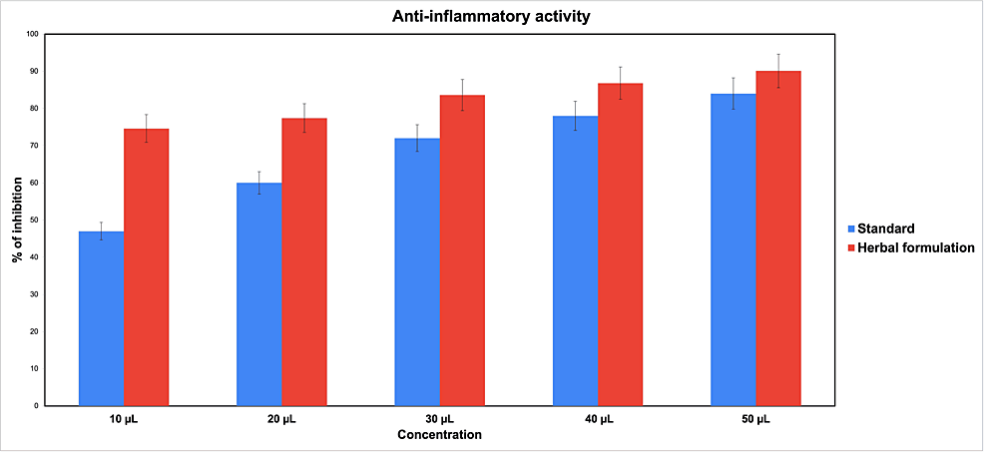
Bar graph showing the anti-inflammatory activity of the gel formulation at various concentrations

The results showed that our plant-based oral gel formulation showed a better anti-inflammatory effect compared to the standard diclofenac used in the study. Different concentrations showed the inhibition of protein denaturation of 74.6, 77.4, 83.6, 86.8, and 90.1 (values in %) (Table 1 and Figure 2) and were comparable with the commercial oral gel with a maximum anti-inflammatory activity of 90.1% at 50 μl concentration. Even at the lowest concentration of 10 μl, the anti-inflammatory activity was shown to be 74.6%. At concentrations of 10 μl, 20 μl, and 30 μl, the anti-inflammatory activity of oral herbal formulations was significantly more than the control (p<0.05); the activity was better at 40 μl and 50 μl also; however, the results were not statistically significant. 

**Table 1:** Comparative analysis of the anti-inflammatory property of the prepared formulation with control; a p-value of less than or equal to 0.05 was considered significant

Concentration	Standard	Herbal formulation	p-value
10 µL	47%	74.6%	0.053
20 µL	60%	77.4%	0.07
30 µL	72%	83.6%	0.057
40 µL	78%	86.8%	0.062
50 µL	84%	90.1%	0.053

Antioxidant activity

The antioxidant assay of the herbal gel formulated was done using free radical DPPH. Methanolic violet-colored DPPH is reduced by hydrogen or electrons to a yellow- or non-colored solution. Table 2 demonstrates the radical scavenging activity of the *C. nucifera* and *T. aestivum* gel formulation at various concentrations. 

**Table 2:** Comparative analysis of the antioxidant property of the prepared formulation with control; a p-value of less than or equal to 0.05 was considered significant

Concentration	Standard	Herbal formulation	p-value
10 µL	76.56%	38.8%	<0.001
20 µL	78.52%	43.2%	0.02
30 µL	85.63%	55.3%	0.012
40 µL	88.68%	54.7%	0.03
50 µL	93.15%	61.3%	0.04

**Figure 3:**
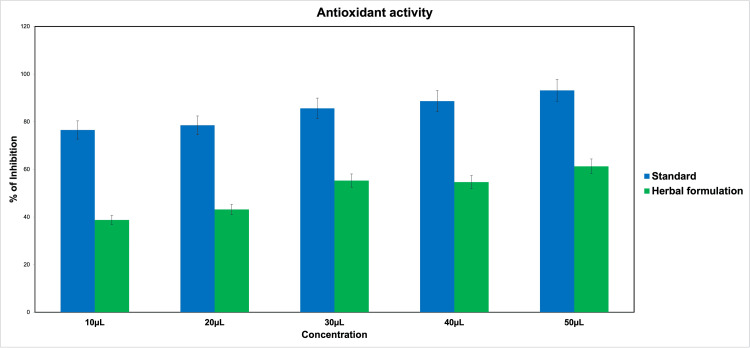
Bar graph showing the antioxidant activity of the oral gel formulation at various concentrations

The percentage of inhibition of the prepared formulation was also noted. At 10 μl, the color changed from yellow to violet with a high scavenging activity of 76.65%, and the highest activity of 99.15% was found to be at 50 μl for standard (Figure 3). The greatest antioxidant activity for the formulated gel was noted at 50 μl. Pertaining to the antioxidant activity, no statistically significant difference was noted between the oral formulation and the control, implying that both showed similar activity (p-value>0.05).

Dose-dependent antioxidant activity was reported to be comparable to ascorbic acid's DPPH scavenging activity.

## Discussion

Studies have demonstrated the effectiveness of plant-derived bioactives in treating oral lesions, including recurrent aphthous stomatitis, mucositis brought on by chemotherapy and radiotherapy, erosive leukoplakia, and oral lichen planus [10]. In this study, the herbal gel formulation was made from the combination of *C. nucifera* and *T. aestivum* extract, and their antioxidant and anti-inflammatory properties were assessed. From the results obtained in our study, the oral gel formulation was found to possess greater antioxidant and anti-inflammatory activities. 

Various studies have reported the medicinal values of the two herbs used in the gel preparation (*C. nucifera* and *T. aestivum*). In a previous study, it was demonstrated that *C. nucifera* and *T. aestivum* oral gel preparation bear significant antimicrobial effects and minimal cytotoxicity [8]. Other studies have evaluated independently the anti-inflammatory and antioxidant properties of *C. nucifera* and *T. aestivum*, but a synergistic effect has not been studied so far. According to Varma et al., coconut oil has been demonstrated to have high anti-inflammatory activity by inhibiting the cytokine level [11]. It was opined that the anti-inflammatory activity of coconut is attributed to its regulatory role in the MAPK signaling pathway, which is one of the key pathways in various cellular and subcellular mechanisms [12].

A variety of phytonutrients, including tannins, phenols, flavonoids, triterpenes, steroids, leucoanthocyanidins, and alkaloids, were found to be present in the ethanolic extract of the coconut mesocarp, whereas the butanol extract of coconut has revealed the presence of condensed tannins, triterpenes, and saponins [13]. In a study by Silva et al., it has been found that the crude extract greatly reduced the amount of paw edema in rats induced by histamine (150 mg/kg) and serotonin (100 and 150 mg/kg). This could be due to the action of coconut on inflammatory mediators or through direct receptor blockage [14]. 

Choudhary et al. demonstrated the increased anti-inflammatory activity of wheatgrass is due to its chlorophyll content [15]. Many clinical studies have recently suggested that wheatgrass has therapeutic benefits for a variety of illnesses [16]. These results were similar to the results of our study in which the anti-inflammatory activity of the gel which is a combination of these two extracts was found to be better than that of the control (diclofenac). As a result, there is a cumulated effect leading to enhanced anti-inflammatory properties of both herbs which could be due to the high amount of polyphenols and flavonoids present in these herbs.

**Figure 4:**
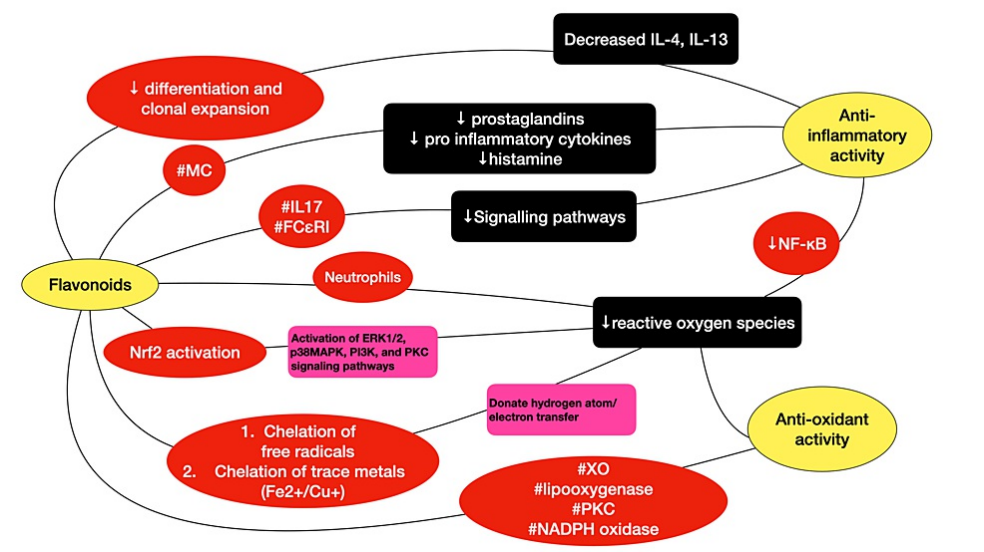
Schematic representation of the pathways involved in flavonoid-mediated anti-inflammatory and antioxidant properties MC: mast cells; IL: interleukin; XO: xanthine oxidase; PKC: protein kinase C; NADP: nicotinamide adenine dinucleotide phosphate; NFkB: nuclear factor kappa B subunit 1; Nrf2: nuclear factor erythroid 2-related factor 2

The antioxidant activity of wheatgrass at various concentrations according to their seed developmental stages was found to have potent antioxidant properties [17]. In an animal study by Mat et al., lipid peroxidation in rats is found to be prevented by ascorbic acid in coconut water, while the other constituent of coconut water, L-arginine, reduces the generation of free radicals [18]. Figure 4 shows a diagrammatic representation of the pathways involved in the anti-inflammatory and antioxidant properties.

This is in concordance with the results obtained in our study, wherein the antioxidant activity of the gel was comparable to the control (ascorbic acid) with the highest concentration of 95% at 50 μl. It was discovered that chlorophyll, which is one of the active ingredients in wheatgrass extract, is what prevents carcinogens from being metabolically activated [16].

The improved antioxidant property may also be attributed to the free radical scavenging mechanism of coconut as demonstrated in various studies [17,19]. Wheatgrass is rich in vitamins which have the ability to scavenge free radicals and are also important components of antioxidant defense systems. This could aid in regulating the release of hydrogen peroxide from the cells. Not only the antioxidant activity but also the high protein and amino acid contents of wheatgrass play a role in cleansing the toxins from the body and possess greater anti-inflammatory activity. In a study by Dasari et al., it has been proven that wheatgrass has the ability to have an anti-inflammatory effect on rat paw edema induced by formalin [20].

Additionally, the high content of vitamins and enzymes which are easily absorbable and other minor mineral elements of both phytomedicines could be attributed to the cumulative enhanced effect of the gel. The antioxidant activity of these phytochemicals has also been emphasized to play a role in chemoprevention by reducing the oxidative stress responsible for the pathogenesis of cancer [21]. 

Considering the numerous medicinal properties of this herbal gel and its biocompatibility, this gel can be well accepted as an alternative oral gel for the treatment of oral lesions and for various other therapeutic purposes. Formulation of such herbal gel with enhanced properties would pave the way for developing herbal alternative medicine which is highly safe and effective. 

Limitations

The standard properties of the gel, like the viscosity test, were not analyzed for the prepared oral gel. In future studies, it is recommended to test the activities of this gel with higher concentrations, and more combinations of herbs can be added and tested for their properties. Another limitation pertains to the confounding bias of not individually testing the anti-inflammatory and antioxidant activities of *C. nucifera* and *T. aestivum*, as we used the combination of these herbs. 

## Conclusions

The combination of these two herbs has been shown to possess potent anti-inflammatory and antioxidant properties. In addition to previously tested good antimicrobial property and low cytotoxicity, the cumulative effects of these two products would aid in the treatment of various oral lesions. The unfavorable side effects of modern medicine have already drawn people's attention to natural remedies. In the future, the usage of more affordable and safer natural products should be employed to compete with contemporary pharmaceuticals. Furthermore, the biological benefits of antioxidant-rich herbs on illnesses linked to oxidative stress require further studies alongside in vivo research and clinical trials.
